# Allergic bronchopulmonary aspergillosis presenting as high-attenuation mucous impaction

**DOI:** 10.1590/0037-8682-0435-2021

**Published:** 2021-11-12

**Authors:** Bruno Hochhegger, Pratik Patel, Edson Marchiori

**Affiliations:** 1 Universidade Federal de Ciências da Saúde de Porto Alegre, Porto Alegre, RS, Brasil.; 2 University of Florida, Florida, USA.; 3 Universidade Federal do Rio de Janeiro, Rio de Janeiro, RJ, Brasil.

**FIGURE 1:**
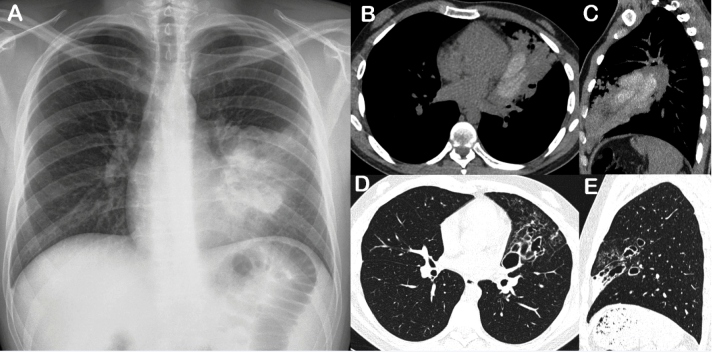
**(A):** Posteroanterior chest radiograph demonstrating heterogeneous opacity in the left lung. Chest CT images with axial **(B)** and sagittal **(C)** reconstruction showing heterogeneous consolidation in the lingula, with branching tubular opacities corresponding to areas of high-attenuation mucoid impaction inside dilated bronchi. **(D, E)** Follow-up CT images obtained 6 months later showing bronchiectasis persistence and resolution of the hyperdense mucous impaction.

A 49-year-old man was admitted to our department with complaints of shortness of breath and wheezing. He had previously received irregular asthma treatment without improvement. A chest radiograph showed a heterogeneous opacity in the left lung (Figure 1A). Chest computed tomography (CT) revealed central bronchiectasis in the lingula with hyperdense mucous impaction (Figures 1B and 1C). Laboratory tests revealed a total serum immunoglobulin E concentration of 2650 IU/ml, eosinophilia (750 cells/mL), and *Aspergillus* skin test positivity. The final diagnosis was allergic bronchopulmonary aspergillosis (ABPA). The patient was treated with oral corticosteroids and itraconazole, which improved his asthma symptoms. Follow-up CT performed six months later showed bronchiectasis persistence and resolution of the hyperdense mucous impaction (Figures 1D and 1E).

ABPA is a complex pulmonary disorder caused by an immune reaction to antigens released by *Aspergillus fumigates,* a fungus that colonizes the tracheobronchial trees of patients with asthma and cystic fibrosis. It presents clinically with refractory asthma, hemoptysis, and systemic manifestations, including fever, malaise, and weight loss. Radiologically, it presents with central bronchiectasis and recurrent episodes of mucoid impaction. The mucus plugs in ABPA are generally hypodense but can be hyperdense on CT. The presence of branching tubular opacities, corresponding to dilated bronchi containing hyperdense mucus, is a characteristic, if not pathognomonic, finding of ABPA^1^ ^-^ ^3^.
